# Moral suasion and charitable giving

**DOI:** 10.1038/s41598-022-24944-6

**Published:** 2022-12-01

**Authors:** Loukas Balafoutas, Sarah Rezaei

**Affiliations:** 1 University of Exeter Business School, Exeter, UK; 2 Department of Public Finance, University of Innsbruck, Innsbruck, Austria; 3 University of Venice “Ca’ Foscari”, Venice, Italy; 4 Department of Economics, Utrecht University, Utrecht, the Netherlands

**Keywords:** Human behaviour, Morality

## Abstract

We investigate the effect of moral suasion on charitable giving. Participants in an online experiment choose between two allocations, one of which includes a donation to a well-known charity organization. Before making this choice, they receive one of several messages potentially involving a moral argument from another participant. We find that the use of consequentialist and deontological arguments has a positive impact on the donation rate. Men respond strongly to consequentialist arguments, while women are less responsive to moral suasion altogether. Messages based on virtue ethics, ethical egoism, and a simple donation imperative are ineffective.

## Introduction

Moral suasion refers to the use of verbal arguments and narratives appealing to morality, with the aim of convincing people to act in a particular way. Governments, charitable and non-profit institutions, and environmental campaigns, among others, widely use moral suasion as an instrument to address global-level challenges like resource conservation, poverty, and climate change. As a result, we are often (e.g., in government announcements or the press) confronted with messages that prompt us to act in line with some moral principle. Evoking such a principle is usually indirect. For instance, during the lockdowns in response to the Covid-19 pandemic, governments worldwide urged their citizens to stay at home, stressing the need to save lives and protect healthcare systems. In addition to such typical utilitarian arguments referring to the greater good of the population, other arguments focused on our duty to protect vulnerable segments of the population, even if the health risks were low to other individuals and despite the possibility that measures against the pandemic come at huge economic costs. These kinds of arguments are more in line with a deontological approach and refer to one’s duties, which are given priority over consequences. A third class of moral arguments concentrated more on a citizen’s character and the need for everyone to assume their personal responsibility in fighting the pandemic, thus echoing the teachings of virtue ethics. Qualitatively, similar kinds of arguments can be found in ongoing debates on the need for vaccination (against a number of diseases), the need for voluntary climate action, the avoidance of antibiotics overuse, charitable giving, and many other situations.

In this paper we present a comprehensive assessment of how different moral narratives and imperatives affect the behavior of experimental participants in a situation that asks them to choose between a selfish and a moral action. In an online experiment, participants play a one-shot dictator game where they are asked to select one of two allocations between themselves and a well-known charity organization (UNICEF). The payoff for the dictator is $3 if they choose option A, and $2 if they choose option B. UNICEF receives $0 if the dictator chooses A, and $3 if they choose option B. Before making their decision, dictators receive (from another subject in the experiment) one of six pre-specified messages. We formulate the messages based on the most influential theories of normative ethics. These messages include (1) a consequentialist (utilitarian) message, which focuses on the consequences of an action and the total benefit that it generates; (2) a deontological message, which focuses on the accordance of an act with a moral duty; (3) a message based on virtue ethics, which (following the tradition of Plato and Aristotle) emphasizes an individual’s moral character. The two allocations in the experiment are designed in a way that donating to UNICEF not only helps someone in need (children in developing countries) but also increases the total sum of payoffs. Hence, deontological, consequentialist, and virtue-based reasoning would support the same course of action. The set of messages also includes, (4) an imperative, i.e., an urge to donate to UNICEF without offering any moral justification; (5) an urge to implement the selfish action based on the reasoning of ethical egoism, the idea that an individual’s sole moral responsibility is to cater for their own well-being^1^, and (6) an empty message as baseline (see experimental instructions in the Supplementary Material for the exact phrasing of the messages).

Taken together, the messages that comprise our experimental variation allow us to address the following question: if the objective is to encourage individuals to behave in a certain pro-social manner, how should one ask? Since different normative moral theories define right and wrong using different arguments, it is an open question which theory is more effective in relative terms. Knowing the answer to this question can be valuable for policy-makers who seek to optimally design their communication with citizens to promote pro-social behavior. Furthermore, it is well-known that men and women differ in many aspects of their behavior, including pro-social preferences^2^. A natural question to ask is, how different are men and women in the way that they respond to moral arguments? An awareness of differential responses by gender may be helpful for policy-makers who want to design messages tailored to the recipient’s gender, paving the way for more personalized interventions.

In answering the above questions, this paper makes two main contributions to the existing literature on moral suasion. First, it measures the effect of different moral appeals on behavior in the form of charitable giving. Economic behavior has been shown to depend on moral preferences^3,4^ and also to respond to moral suasion: for instance, moral arguments been used to promote energy conservation^5,6^, tax compliance^7,8^, TV license fee compliance^9^, organ donation^10^, blood donation^11^, altruistic behavior in the dictator game^12^ and cooperation in public good games^13^. Moreover, there is evidence that pro-social behavior strongly responds to moral nudges, which consist of asking experimental participants about moral norms before they take their decisions^14,15^ . However, we are the first to consider a broad set of messages, including several different narratives and one imperative (following the terminology of Bénabou et al.^16^), and show how they compare in terms of promoting pro-social behavior in the form of donations to charity. Our second main contribution is to study the effect of moral suasion by gender, allowing for differential behavioral responses by men and women are towards different moral messages.

Our work is also related to existing research on the determinants of fair behavior. Previous field and lab experiments reveal that asking people to donate, even without providing any reasons, often increases donation rates^17,18^. Other key factors that have been shown to increase generosity in experimental settings include reduced social distance^19,20^, recipient deservingness^21^ and recipient poverty^22^. Moreover, increased visibility has been shown to induce moral behavior^23,24^. There is a relationship between fair behavior and the desire to maintain a positive identity or a person’s sense of self^25–27^. Bénabou et al.^16^ show that providing people with excuses and possibilities to justify their actions affects their pro-social behavior. In our experiment, apart from moral messages that ask people to donate, a group of participants receives a message encouraging them to be selfish, while another group receives no message. Hence, our framework includes both exculpatory and responsibilizing messages.

Although moral suasion is widely used by charity organizations, economists have not yet systematically studied its effects on giving to charities. Some loosely related research has studied the impact of directly asking for a certain amount of money^28^ and the impact of communication on giving^17,18^, showing that “asking is powerful” and giving increases when people are explicitly asked to do so. Moreover, framing has been shown to affect participants’ charitable behavior in lab and field experiments. For instance, a stream of research shows that changing the names of strategies of a game or the name of the game can affect decision makers’ choices^12,29–33^.

A substantial body of literature has shown that males and females have different preferences for resource distribution. For instance, Croson and Gneezy^2^ report that women are more inequality averse and favor equality in giving. Regarding gender differences in altruism, while some studies find no gender difference^34^, most studies on the topic report that women are more generous than men^35–38^. Moreover, females appear to favor equity over efficiency more than males do^39^. As a result, they respond more favorably to calls for help, while men respond more to their personal benefits of giving^40,41^. This is in line with another research strand in the field of moral judgment, showing that women tend to embrace deontological ethics more than men^42^, while men tend to embrace consequentialism^43^ in personal dilemmas. The difference between impersonal and personal moral dilemmas comes from the extent to which a participant is emotionally involved^44^.

Our results provide evidence that moral suasion can be effective in increasing donation rates, but its effectiveness depends on the message used and on the gender of the message recipient. Overall, the deontological message is the most successful in encouraging participants to donate, increasing donation rates by 19%, followed by the consequentialist message with a somewhat smaller (14%) and weakly significant effect. The other messages (based on virtue ethics, ethical egoism, and a simple imperative) have no significant impact on behavior in our experiment. Turning to the relationship between moral suasion and gender, we find that the female participants have a higher baseline willingness to donate, and they appear to be less sensitive to messages than their male counterparts. While the highest rate of donations among male participants belongs to the group that received the consequentialist message, female participants are most likely to donate after receiving the deontological message.

## Experiment

We ran an online experiment with 32 sessions conducted in two waves between June 2021 and January 2022 in Amazon Mechanical Turk (henceforth MTurk). The experiment was conducted using oTree^45^, and we recruited a total of 2426 participants between 18 and 88 years ($$M=37.38$$, $$SD=11.08$$). The experiment lasted on average 15 minutes, with average payments of $2.59 per subject with a minimum of $2.1 and a maximum of $3.1, including a $0.1 participation fee, besides the payments made to UNICEF, which amounted to $3309 in total.

Experimental participants in all sessions acted as dictators in a simple donation game, where they were asked to choose between two possible monetary allocations for themselves and UNICEF. Specifically, they could choose between option A, resulting in $3 to themselves and $0 to UNICEF, and option B, resulting in $2 to themselves and $3 to UNICEF. Hence, one of the options (A) can be considered selfish since it maximizes the participant’s payoff but leaves nothing on the table for charity, while the other option (B) can be considered altruistic because participants who choose it are willingly giving up $1 in order to generate a donation worth $3. Before making their decision, participants received a message sent by another Mturk participant who was familiar with the game but could not play it herself (see the experimental instructions for message receivers and senders in the Supplementary Material). This message varied across participants in a between-subjects treatment variation, which consisted of six treatments (messages) in total. For the remainder of this paper, we will thus be using the terms “message” and “treatment” interchangeably.

The messages that experimental participants received from the sender included three messages following consequentialism, deontology, and virtue ethics, which—in this binary donation game—all prescribe that the participants donate to UNICEF. One message follows the logic of ethical egoism that appeals to self-interested behavior and suggests the participants keep the money for themselves. One imperative message asks the participant to donate to UNICEF without any explanation. Finally, we implemented a baseline treatment of no message, where the recipient is informed that the sender did not send him any message. Below we list these messages:

1. *Consequentialism* Donating to UNICEF is the moral course of action because it helps the ones in need and maximizes the total welfare (sum of everyone’s payoff) you generate. So, if you consider the consequences of your action, you should donate.
2. *Deontology* Donating to UNICEF is the moral course of action because it is everyone’s duty to help the ones in need. So, if you consider your moral duty, you should donate.
3. *Virtue ethics* Donating to UNICEF is the moral course of action because a good and virtuous person would show generosity and help the ones in need. So, if you consider the importance of being virtuous, you should donate.
4. *Ethical egoism* Donating nothing to UNICEF is the right course of action because it is everyone’s responsibility to look after themselves and maximize their own income, rather than the income of others.
5. *Imperative* You should donate to UNICEF.
6. *No message* –

At the end of the experiment, participants answered two questionnaires. One questionnaire included nine demographic questions, and the other one was the 20 item version of the Moral Foundations Questionnaire^46,47^. This questionnaire measures the degree to which people prioritize five fundamental domains in moral decision-making: Harm/Care, Fairness/Reciprocity, In-group/Loyalty, Authority/Respect, and Purity/Sanctity. These moral foundations have been linked to deontological and consequentialist ethics in the context of persuasive moral communication^48^.

To avoid deception, in a separate session (in June 2021) we recruited 61 Mturk workers to play the role of the message sender. These subjects were informed about the game that the recipients of the messages would play. They could not play the donation game themselves, but they could send a message to the participants who would play it. The message had to be selected from the list of six pre-specified options shown above . The experiment lasted for about 10 minutes on average, and subjects received a fixed payment of $2.1 including a $0.1 participation fee independent of the message they sent.

This research received approval from the Board for Ethical Questions in Science of the University of Innsbruck, and informed consent was obtained from all participants. All experiments were performed in accordance with the guidelines and regulations set out by the ethics committee. Before starting with the data collection, we preregistered our research design and analyses on AsPredicted [see pre-registrations 1 (https://aspredicted.org/64kb7.pdf) and 2 (https://aspredicted.org/a873a.pdf)]. The first of those pre-registrations describes the overall effect of moral suasion on donations, while the second also considers the effects on male and female participants separately (following initial reactions to our manuscript and concerns about sample size). Since the experiments are identical, and in order to benefit from a higher statistical power, we merge the data of the two experiments in the analysis.

## Data description

We imposed three restrictions on participation in the experiment: workers had to be registered in the U.S., have an internal MTurk approval rating for past tasks of over 95%, and have at least 100 completed tasks. We allowed every worker to participate at most once and excluded workers who did not complete the entire experiment.

**Table 1:** Summary statistics by message.

	All	C	D	V	E	I	B	*p* value
*Male*	0.50 (0.50)	0.50 (0.50)	0.50 (0.50)	0.47 (0.50)	0.54 (0.50)	0.51 (0.50)	0.49 (0.50)	0.477
*Age*	37.38 (11.09)	37.49 (11.31)	37.63 (10.51)	38.02 (11.44)	37.21 (11.12)	37.02 (10.87)	36.94 (11.23)	0.686
*White*	0.81 (0.39)	0.81 (0.39)	0.79 (0.41)	0.82 (0.38)	0.81 (0.40)	0.80 (0.40)	0.82 (0.38)	0.803
*Highly educated*	0.91 (0.29)	0.92 (0.27)	0.89 (0.31)	0.91 (0.29)	0.93 (0.26)	0.88 (0.32)	0.91 (0.29)	0.193
*Savings*	1.91 (0.99)	2.01 (1.03)	1.89 (1.00)	1.96 (1.00)	1.91 (0.98)	1.88 (0.96)	1.82 (0.98)	0.103
*Social class*	2.78 (0.92)	2.82 (0.92)	2.84 (0.90)	2.82 (0.91)	2.74 (0.90)	2.78 (0.97)	2.72 (0.90)	0.382
*Political view*	6.94 (2.55)	6.83 (2.60)	6.89 (2.52)	6.77 (2.59)	6.92 (2.62)	6.98 (2.61)	7.26 (2.32)	0.168
*Moral conviction*	6.36 (2.59)	6.49 (2.65)	6.04 (2.53)	6.29 (2.61)	6.55 (2.59)	6.18 (2.71)	6.58 (2.44)	0.009
*Religious*	1.35 (0.59)	1.33 (0.60)	1.32 (0.56)	1.35 (0.60)	1.39 (0.64)	1.38 (0.60)	1.30 (0.55)	0.162
*Harm/Care*	3.86 (0.78)	3.85 (0.74)	3.83 (0.77)	3.84 (0.82)	3.89 (0.77)	3.92 (0.80)	3.82 (0.75)	0.209
*Fairness/Cheating*	3.87 (0.74)	3.88 (0.74)	3.84 (0.79)	3.85 (0.75)	3.92 (0.69)	3.89 (0.78)	3.86 (0.68)	0.646
*In-group/Loyalty*	3.42 (1.07)	3.44 (1.03)	3.41 (1.07)	3.34 (1.09)	3.45 (1.08)	3.38 (1.14)	3.47 (0.99)	0.671
*Authority/Respect*	3.46 (1.02)	3.43 (1.03)	3.49 (0.98)	3.42 (1.04)	3.45 (1.03)	3.48 (1.07)	3.49 (0.95)	0.780
*Purity/Sanctity*	3.38 (1.09)	3.35 (1.11)	3.43 (1.03)	3.31 (1.07)	3.35 (1.14)	3.41 (1.16)	3.44 (1.00)	0.393
$N$	2426	419	385	401	423	386	412	

Means reported, standard deviations in parentheses. Column abbreviated names refer to *Consequentialism* (C), *Deontology* (D), *Virtue ethics* (V), *Ethical egoism* (E), *Imperative* (I) and *Baseline* (B). The p-values correspond to Kruskal-Wallis equality-of-populations rank test for continuous variables, and $\chi ^{2}$ tests for binary variable (i.e. Male, White, and Highly educated).

Table 1 provides the summary statistics of the experiment across all messages and divided by the type of messages. *Male* is a dummy variable indicating whether the participant is a male. *Age* is a continuous variable indicating age. White is a dummy variable indicating the worker self-identifies as white. *Higher educated* is a dummy variable indicating the worker completed at least some college at the undergraduate level. We also report a few questions from the *World Values Survey* (WVS) measuring family *savings* during the past year, ranging from 1 (saved money) to 4 (spent savings and borrowed money), *social class* ranging from 1 (upper class) to 5 (lower class), the strength of *moral convictions* ranging from 1 to 10, *religious* values ranging from 1 (a religious person) to 3 (an atheist), and *political view* ranging from 1 (far left) to 10 (far right). Finally, *Harm/Care*, *Fairness/Cheating*, *In-group/Loyalty*, *Authority/Respect*, and *Purity/Sanctity* are indices capturing the five morality dimensions based on the *Moral Foundations Questionnaire* (or MFQ). The minimum score for each value is 0, and the maximum is 5.

Table 1 shows that about half of the participants are male, and more than 80% are white. The average age is around 37, and 90% of the participants have some undergraduate education. The table indicates that randomization has been successful: out of the 14 variables presented in the table, only one (moral convictions) is not balanced across treatments (based on the p values reported in the last column). Our regression analysis includes specifications that control for all available participant characteristics.

## Results

### Effect of moral suasion on donations

**Figure 1:**
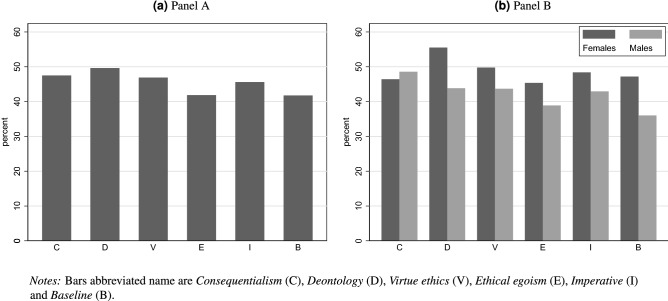
Donation rate across messages.

Over the entire experiment, 1103 out of 2426 participants $(45.5\%)$ chose to donate. To measure the effect of different messages on donations, we begin by looking at the percentage of participants who chose to donate to UNICEF in each treatment (message group). Panel A in Fig. 1 shows that there are differences in donation rates across messages. Participants are, on average, more likely to donate if they receive one of the three moral messages: *consequentialism*, *deontology* or *virtue ethics*. The highest rate of donation belongs to the participants who received the *deontology* message with a donation rate of $49.6\%$. The lowest donation rates belong to the baseline and *ethical egoism*, with donation rates of $41.7\%$ and $41.8\%$, respectively.

Pairwise comparisons in donation rates against the baseline treatment of *no message* allow us to formally test for the presence of moral suasion. Using $\chi ^{2}$ tests of independence for these comparisons, we show that donation rates for those who received the *consequentialism* and *deontology* messages are higher than in the baseline. This difference is larger with the deontological message ($49.6\%$ vs. $41.7\%$, $p=0.026$; $\chi ^{2}$ test), and it is weakly significant in the case of the *consequentialist* message ($47.5\%$ vs. $41.7\%$; $p=0.096$). There is no significant difference effect of any of the remaining messages (*virtue ethics*, *ethical egoism*, *imperative*) on donations ($p=0.141$, 0.977, 0.273, respectively).

The use of *no message* as a baseline might, in principle, overestimate treatment differences if some participants perceive the empty message as encouraging selfish behavior (because it reveals that the sender decided not to engage in moral suasion). To address this concern, we ran an additional treatment on Mturk (*N*=200) as a robustness test. Participants in this case were asked to make the same donation choice as in all other treatments, but this time there was no sender (and no message) involved. We find that donation rates were, in fact, *lower* in this case compared to our main baseline treatment ($34.0\%$ vs. $41.7\%$; $p=0.066$). Hence, if anything, our choice of baseline leads to a conservative estimate of the effects of moral messages on donations.

Next, following our preregistration, we compare donations rates across messages broken down by the participants’ gender. Panel B of Fig. 1 shows the rate of donation to UNICEF across different messages, separately for male and female participants. Visual inspection of this figure reveals that male and female participants respond differently to the messages considered in this experiment. Men have a relatively low baseline willingness to donate (of $36.0\%$), but donation rates are higher with every message (including the message that prompts participants to choose the selfish option). However, the only message that significantly impacts donation rates among male participants is the consequentialist one, which brings donations by men up to $48.6\%$. This represents a very sizeable increase of $34.9\%$ relative to the baseline ($p=0.010$).

The pattern substantially changes when we consider female participants only. We begin by noting that, in line with much of the literature on gender and giving, women display much higher donation rates than men in the absence of any message ($47.2\%$ vs. $36.0\%$, $p=0.022$). For women, the message that appears to have the strongest effect on charitable giving is the deontological one, which increases donation rates among female participants from $47.2\%$ to $55.5\%$ ($p=0.095$). On the contrary, the *imperative*, *ethical egoism*, *virtue ethics*, and *consequentialism* messages are all associated with negligible (and insignificant) differences compared to the baseline.

We conclude this part of the analysis by briefly comparing the means of the moral foundation variables across gender. Female participants score significantly higher than their male counterparts in two dimensions. The first is *Harm/Care* (3.92 vs. 3.79, $p= 0.0001$), which according to the moral foundations theory is associated with empathy and kindness. This is in line with the higher donations of women in the baseline condition, and also with previous research showing that women are on average more altruistic than men and more averse to distributional inequalities. The second dimension where female participants score higher is *Fairness/Cheating* (3.91 vs. 3.84, $p= 0.019$). The size and the sign of the differences between male and female participants in these two dimensions are similar across all the messages, and there is no statistically significant difference between male and female participants in any of the other dimensions (In-group/Loyalty, Authority/Respect, Purity/Sanctity).

### Regression analysis

**Figure 2:**
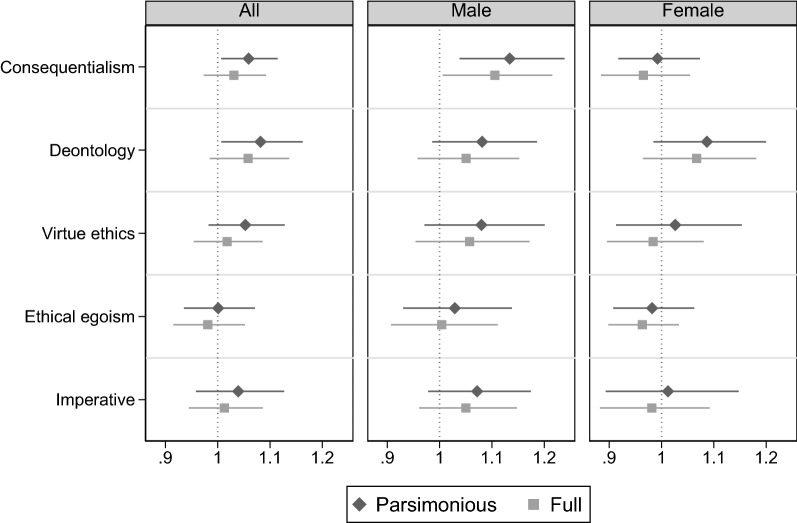
Relationship between moral messages and donation rates, pooled and by gender.

Our treatments are designed to identify the causal effects of moral suasion on donation rates. Figure 2 plots the relationship between the moral messages and participants’ choice to donate. Each relationship is estimated with an OLS regression, with the donation rate as the dependent variable. “Parsimonious” points plot the coefficients from an OLS regression of donation on dummies for all five messages (with the baseline as the omitted category). In contrast, “Full” points plot the coefficients when the regression specification additionally includes the full set of available control variables (i.e., demographics, religious and political attitudes, moral foundations). Bars indicate $95\%$ confidence intervals. The reference group in all these regressions is the participants who received the baseline of *no message*. To account for the fact that the data were collected on several different days, we cluster standard errors by session date in all specifications. Tables SM.1, SM.2 and SM.3 in Supplementary Material provide the complete regression tables.

Figure 2 highlights several findings. First, as discussed earlier, the *consequentialism* and *deontology* messages have a positive and statistically significant effect on the donation rate in the pooled sample in the parsimonious specification (see panel “All” and also columns 1 and 2 in Table SM.1 in the Supplementary Material), while the effect of the remaining messages is small and insignificant. The biggest effect size of a message belongs to *deontology*, which leads to an increase of over 7 percentage points in the likelihood that a participant donates. Both these effects become weaker as more control variables are added and end up losing significance in the full regression that controls for all available individual characteristics. This indicates that a substantial part of the correlation between donation rates and messages can be attributed to these characteristics.

Second, the disaggregated regressions by gender confirm the qualitatively different moral suasion patterns for male and female participants. The differences across messages are not particularly large for female participants, and none of them is significant at the 5% significance level. Panels “Male” and “Female” in Fig. 2 show that the positive effect of *consequentialism* on the donation rate is entirely driven by male participants, for whom it is of a remarkable magnitude exceeding 10 percentage points (see also Table SM.2 in the Supplementary Material ). Adding the control variables (leading to the “Full” coefficients) does not substantially change the magnitude of the coefficients for male participants, indicating that the inclusion of participant characteristics does not increase the model’s overall precision and explanatory power. For women, the strongest increase in donation rates comes through the *deontology* message, which, however, is only marginally significant and only in the parsimonious specification. The effect of all the other messages on the likelihood of choosing to donate is practically zero. Hence, we conclude that we find only limited evidence of women being motivated by deontological arguments. The overarching pattern for women seems to be that their behavior does not respond to moral suasion.

Finally, we examine whether receiving a particular message has a different effect on men and women. The coefficients of the relevant interaction terms reported in Table 2 capture the differential effects by gender, once based on a parsimonious specification and once including the full set of controls (demographics, socioeconomic status, and moral foundations), and clustering the standard errors by date. The only message that is found to have a (weakly) significantly different effect for men and women is the consequentialist one, which—as the previous analysis has shown—leads to a strong increase in the willingness to donate among male participants.

We note that we have estimated all regressions using the Probit model instead of OLS and confirm that all main findings remain qualitatively the same. Tables SM.4, SM.5, SM.6 and SM.7 in the Supplementary Material provide complete Probit regression tables and Figure SM.1 plots the relationship between the moral messages and participants’ choice to donate using Probit regressions.

**Table 2:** Gender differences in the effects of messages.

	(1)	(2)
**Dependent variable: donation to UNICEF**		
Consequentialism$\times$ male	0.104* (0.057)	0.103* (0.055)
Deontology$\times$ male	$-$ 0.062 (0.046)	$-$ 0.078 (0.050)
Virtue ethics $\times$ male	0.004 (0.076)	0.029 (0.061)
Ethical egoism$\times$ male	$-$ 0.002 (0.052)	$-$ 0.015 (0.059)
Imperative $\times$ male	0.012 (0.052)	0.014 (0.047)
Baseline $\times$ male	$-$ 0.056 (0.050)	$-$ 0.052 (0.046)
Additional controls	No	Yes

Coefficients from OLS models (standard errors in parentheses) and controlling for the date of data collection. Additional controls are age, ethnicity, political view, moral conviction and the five MFQ moral values. *$p<0.1$; **$p<0.05$, ***$p<0.01$.

## Discussion and conclusion

We have chosen to work with an experimental game in which a particular outcome (donating to charity) is unambiguously endorsed by *consequentialism*, *deontology*, and *virtue ethics*. This choice of game is associated with two important caveats. First, moral judgment in reality is often cast within dilemmas, for instance when tensions arise between utilitarian and deontological considerations (as in cases resembling the paradigmatic trolley dilemma). Our design is not equipped to study moral suasion in such cases, because our objective has been to assess the relative effectiveness of different moral messages in achieving the same (shared) goal.

Second, we cannot know with certainty whether and how our main findings on moral suasion extrapolate to other types of games, although recent evidence suggests that the effect of moral nudges can persist across contexts^14^. In this respect, a comparison with some key related experimental work can also be helpful. Our finding that the strongest effect in the pooled sample is achieved by means of the consequentialist message, which makes the utilitarian point that aggregate welfare is higher when participants donate, is in line with previous related empirical evidence. Aguiar et al.^49^ ask subjects to self-report their motivations for donating to charity, and find that consequentialist reasons are the most prominent ones (accounting for about 60 percent of responses). Dal Bó and Dal Bó^13^ study the effect of moral suasion in a public goods game played over 20 rounds. They show that the so-called ‘golden rule’ (asking people to treat others the way they want to be treated, a doctrine going back at least to the teachings of Confucius), as well utilitarian messages, resulting in a significant but transitory increase in contributions compared to a baseline of no message and to a selfish message (which is similar to our ethical egoism treatment). When players have the option of punishing each other after the contribution stage, the effect of the moral messages on contributions becomes more persistent. Consistent with our results, Hillenbrand and Verrina^15^ find that a message encouraging experimental participants to act selfishly (a “negative narrative” has no influencfe on behavior, while positive narratives can increase dictator giving. Capraro and Sippel^42^ explore gender differences in a vignette study with three moral dilemmas: a typical personal dilemma, a typical impersonal dilemma, and an intermediate dilemma. They show that women tend to embrace deontological ethics more than men in personal, but not impersonal, dilemmas. They find no gender differences in the intermediate situation. These findings align with a key pattern we observe in our data, namely that women are more responsive than men to moral persuasion based on deontology.

The aim of this paper has been to contribute to the literature on moral suasion by examining, within the same framework, the effects of different messages on donation rates in a preregistered online experiment. We have found that moral suasion can be effective if it uses consequentialist and deontological arguments, while messages based on virtue ethics and a simple imperative that is not supported by moral arguments are ineffective. We have also included a message encouraging participants to act selfishly in line with ethical egoism to see whether providing an exculpatory message will lead people to behave more selfishly. This is not the case. We note, nevertheless, that the effectiveness of imperatives in real-world settings is likely to depend on the authority of the individual issuing them^16^. We have disaggregated the analysis by gender and documented different patterns, with men responding strongly to consequentialist messages and women showing a much more modest response altogether. We believe that these results can inform policy-makers who are looking for effective ways of persuading individuals to act in line with a given pro-social maxim, for instance, in the context of collective climate action or vaccination campaigns. Our finding that moral suasion differs by gender can be particularly useful when designing targeted personalized interventions.

## Supplementary Information

Supplementary Information.

## Data Availability

The datasets generated during and/or analyzed during the current study are available from the corresponding author on reasonable request.
