# Supplementary material for: Moral suasion and charitable giving
Source: Sci Rep. 2022 Dec 1;12:20780. doi: 10.1038/s41598-022-24944-6 (PMC9714400; doi:10.1038/s41598-022-24944-6)
Supplement: Supplementary file 1 — Supplementary Information. [file 41598_2022_24944_MOESM1_ESM.pdf]

Supplementary Information for “Moral Suasion and Charitable Giving”

Loukas Balafoutas[1] **and Sarah Rezaei**[2]

[1]University of Exeter Business School, Exeter, UK; University of Innsbruck, Department of Public Finance, Innsbruck, Austria and University of Venice “Ca’ Foscari”, Venice, Italy
[2]Utrecht University, Department of Economics, Utrecht, the Netherlands and University of Innsbruck, Department of Public Finance, Innsbruck, Austria

Supplementary Information

Part I: Experimental instructions

Instructions for the receivers of moral messages

Welcome to this experiment!

This Human Intelligence Task (HIT) is an experiment on decision-making. It will require you to perform a few simple tasks. Your payment will be based on your performance. We also ask some survey questions after the tasks. All data will be stored anonymously. The entire HIT takes around 10 minutes to complete, and payment is conditional on full completion of the tasks and survey: please only accept this HIT if you can commit to completing it. You have 60 minutes to complete this HIT. After completing this HIT, you will earn 0.1 US dollars for participating, and up to 3.0 US dollars in total depending on your performance.

After you have accepted this HIT, the URL to the study will appear here: link.

On the last page, you will be given a completion code. Please copy/paste that code below.

Your partner and decisions

As part of this experiment, a partner has been randomly assigned to you. This partner (henceforth Participant B) is another participant in this experiment.

On the following screen, you will be asked to make one decision: You will have to choose between two possible monetary allocations for yourself and the charity organization UNICEF. Any amount of money you keep for yourself will be paid to you at the end of the experiment. Any amount you allocate to UNICEF will be donated to UNICEF at the end of the experiment.

Before you make your decision, we present the same allocation task to Participant B. Participant B cannot make the same decision as you, but he (she) will be asked to send you a message. Then, we present his (her) message to you.

Summing up, here is the timeline of the experiment:

- Participant B decide which message to send to you.
- You see Participant B’s message and make your decision on an allocation between yourself and UNICEF.
- You complete a questionnaire.

Note: Click on ”Next” when you have carefully read through the information. You can only click on the ”Next” button if you have spent at least 60 seconds on this page.

Information about UNICEF

The following information is important and relevant to the decisions you are going to make. Please read through all the information carefully.

UNICEF works in the world’s toughest places to reach the most disadvantaged children and adolescents – and to protect the rights of every child everywhere. Across more than 190 countries and territories, we do whatever it takes to help children survive, thrive, and fulfill their potential from early childhood through adolescence. The world’s largest provider of vaccines, we support child health and nutrition, safe water and sanitation, quality education, and skill-building, HIV prevention and treatment for mothers and babies, and the protection of children and adolescents from violence and exploitation. Before, during, and after humanitarian emergencies, UNICEF is on the ground,

bringing lifesaving help and hope to children and families. Non-political and impartial, we are never neutral when it comes to defending children's rights and safeguarding their lives and futures. For more information, see here.

What UNICEF does with this specific donation?

With this amount, for example:

- UNICEF gives three young children therapeutic milk against malnutrition.
- UNICEF provides measles vaccinations for five children.
- Three children can receive ORS treatment, which prevents dehydration due to diarrhea.

Click on Next when you have carefully read through the information. You can only click on the "Next" button if you have spent at least 40 seconds on this page.

Decision

As already explained previously, below you are asked to choose between two possible allocations, called Option A and Option B. The two options are as follows:

- Option A: $3 to you and $0 to UNICEF
- Option B: $2 to you and $3 to UNICEF

We have also described these options to Participant B, who cannot take any action at all. All Participant B could do was send you a message. Below is the message that Participant B has sent to you:

> Donating to UNICEF is the moral course of action because it is everyone's duty to help the ones in need. So, if you consider your moral duty, you should donate."

Choose one of the options below:

☐ Option A: $3 to you and $0 to UNICEF ☐ Option B: $2 to you and $3 to UNICEF

Instructions for the senders of moral messages

Please note that the pages "Instruction" and "Information about UNICEF" are the same as the ones of the receivers of the moral messages.

Your partner and decisions

As part of this experiment, a partner has been randomly assigned to you. This partner (henceforth Participant B) is another participant in this experiment.
Participant B will have to choose between two possible monetary allocations for himself (herself) and the charity organization UNICEF. Any amount of money he (she) keeps for himself (herself) will be paid him (her) at the end of the experiment. Any amount he (she) allocates to UNICEF will be donated to UNICEF at the end of the experiment.
On the following screen you will be asked to make one decision:
You will be asked to select a message from a list of pre-written messages to send to Participant B. Participant B receives your message before choosing between two possible monetary allocations.
Please note that your payoff from this task is $2, and it does not depend on your message or Participant B's choice.
After you have made these decisions, you will be asked to complete a questionnaire that will consist of two screens. Then, the experiment will end.
Summing up, here is the timeline of the experiment:

- You decide which message to send to Participant B.
- Participant B sees your message, and makes his (her) decision on an allocation between himself (herself) and UNICEF.
- You complete a questionnaire.

Click on ”Next” when you have carefully read through the information. You can only click on the “Next” button if you have spent at least 45 seconds on this page.

Decision

As already explained previously, Participant B will soon have to choose between two possible allocations, called Option A and Option B. The two options are as follows:

- Option A: $3 to you and $0 to UNICEF
- Option B: $2 to you and $3 to UNICEF

Before Participant B makes the decision, you can send him (her) a message. Please choose the message that you would like to send to participant B.

☐ Donating to UNICEF is the moral course of action because it helps the ones in need and maximizes the total welfare (sum of everyone’s payoff) you generate. So, if you consider the consequences of your action, you should donate.

☐ Donating to UNICEF is the moral course of action because it is everyone’s duty to help the ones in need. So, if you consider your moral duty, you should donate.

☐ Donating to UNICEF is the moral course of action because a good and virtuous person would show generosity and help the ones in need. So, if you consider the importance of being virtuous, you should donate.

☐ Donating nothing to UNICEF is the right course of action because it is everyone’s responsibility to look after themselves and maximize their own income, rather than the income of others.

☐ You should donate to UNICEF.

☐ -

Part II: Regressions

Linear regression models

Table SM.1. The impact of moral messages on donation rate

	(1)	(2)	(3)	(4)	(5)	(6)	(7)	(8)
consequentialism	0.058**	0.057**	0.033	0.047*	0.033	0.047*	0.031	0.031
	(0.02)	(0.02)	(0.03)	(0.03)	(0.03)	(0.03)	(0.03)	(0.03)
Deontology	0.079**	0.073**	0.059	0.075**	0.056	0.071**	0.059*	0.056
	(0.03)	(0.03)	(0.04)	(0.03)	(0.04)	(0.03)	(0.03)	(0.04)
Virtue ethics	0.051	0.046	0.025	0.037	0.021	0.033	0.021	0.018
	(0.03)	(0.03)	(0.03)	(0.03)	(0.03)	(0.03)	(0.03)	(0.03)
Ethical egoism	0.001	0.004	-0.015	-0.013	-0.012	-0.010	-0.022	-0.019
	(0.03)	(0.03)	(0.03)	(0.03)	(0.03)	(0.03)	(0.03)	(0.03)
Imperative	0.039	0.036	0.023	0.022	0.023	0.023	0.0122	0.013
	(0.04)	(0.04)	(0.04)	(0.04)	(0.04)	(0.04)	(0.03)	(0.03)
Constant	0.417***	9.518***	0.626***	0.249***	9.384***	7.808***	0.297***	8.017***
	(0.02)	(2.91)	(0.05)	(0.08)	(2.62)	(2.57)	(0.07)	(2.40)
Control for demographics	No	Yes	No	No	Yes	Yes	No	Yes
Control for MFQ	No	No	No	Yes	No	Yes	Yes	Yes
Additional controls	No	No	Yes	No	Yes	No	Yes	Yes
N	2426	2426	2426	2426	2426	2426	2426	2426

Notes: Coefficients from linear regression models. Standard errors in parentheses, clustered by date of data collection. $^{*}p < 0.1$, $^{**}p < 0.05$, $^{***}p < 0.01$.

Table SM.2. The impact of moral messages on male donation rates

	(1)	(2)	(3)	(4)	(5)	(6)	(7)	(8)
consequentialism	0.126***	0.128***	0.103**	0.114**	0.103**	0.115**	0.101**	0.100**
	(0.04)	(0.04)	(0.05)	(0.05)	(0.05)	(0.05)	(0.05)	(0.05)
Deontology	0.078*	0.075	0.059	0.066	0.053	0.061	0.055	0.049
	(0.05)	(0.05)	(0.05)	(0.05)	(0.05)	(0.05)	(0.05)	(0.05)
Virtue ethics	0.077	0.077	0.056	0.072	0.055	0.072	0.057	0.056
	(0.05)	(0.05)	(0.05)	(0.05)	(0.05)	(0.05)	(0.05)	(0.05)
Ethical egoism	0.029	0.030	0.006	0.017	0.006	0.017	0.005	0.004
	(0.05)	(0.05)	(0.05)	(0.05)	(0.05)	(0.05)	(0.05)	(0.05)
Imperative	0.069	0.070	0.055	0.056	0.056	0.059	0.047	0.049
	(0.04)	(0.05)	(0.05)	(0.05)	(0.05)	(0.05)	(0.05)	(0.05)
Constant	0.360***	6.644*	0.539***	0.358***	7.939**	6.958**	0.381***	7.476**
	(0.03)	(3.30)	(0.07)	(0.11)	(3.21)	(3.02)	(0.09)	(2.96)
Control for demographics	No	Yes	No	No	Yes	Yes	No	Yes
Control for MFQ	No	No	No	Yes	No	Yes	Yes	Yes
Additional controls	No	No	Yes	No	Yes	No	Yes	Yes
N	1221	1221	1221	1221	1221	1221	1221	1221

Notes: Coefficients from linear regression models. Standard errors in parentheses, clustered by date of data collection. $^{*}p < 0.1$, $^{**}p < 0.05$, $^{***}p < 0.01$.

Table SM.3. The impact of moral messages on female donation rates

	(1)	(2)	(3)	(4)	(5)	(6)	(7)	(8)
consequentialism	-0.008	-0.022	-0.031	-0.013	-0.038	-0.023	-0.030	-0.0350
	(0.04)	(0.04)	(0.04)	(0.04)	(0.04)	(0.04)	(0.04)	(0.04)
Deontology	0.083*	0.071	0.067	0.092*	0.059	0.081	0.072	0.065
	(0.05)	(0.05)	(0.05)	(0.05)	(0.05)	(0.05)	(0.05)	(0.05)
Virtue ethics	0.026	0.013	-0.005	0.001	-0.011	-0.005	-0.013	-0.016
	(0.06)	(0.05)	(0.05)	(0.05)	(0.05)	(0.05)	(0.05)	(0.05)
Ethical egoism	-0.018	-0.020	-0.022	-0.036	-0.025	-0.036	-0.036	-0.037
	(0.04)	(0.04)	(0.04)	(0.03)	(0.04)	(0.03)	(0.03)	(0.03)
Imperative	0.012	-0.002	-0.003	-0.004	-0.012	-0.012	-0.013	-0.019
	(0.06)	(0.06)	(0.06)	(0.06)	(0.05)	(0.06)	(0.05)	(0.05)
Constant	0.472***	11.50***	0.714***	0.125	9.900***	7.619**	0.203	7.340**
	(0.04)	(3.93)	(0.08)	(0.10)	(3.42)	(3.63)	(0.127)	(3.307)
	(0.03)	(2.52)	(0.07)	(0.10)	(2.51)	(2.50)	(0.11)	(2.51)
Control for demographics	No	Yes	No	No	Yes	Yes	No	Yes
Control for MFQ	No	No	No	Yes	No	Yes	Yes	Yes
Additional controls	No	No	Yes	No	Yes	No	Yes	Yes
N	1205	1205	1205	1205	1205	1205	1205	1205

Notes: Coefficients from linear regression models. Standard errors in parentheses, clustered by date of data collection. $^{*}p < 0.1, ^{**}p < 0.05, ^{***}p < 0.01$.

Probit models

Figure SM.1. Relationship between moral messages and donation rates, pooled and by gender (using Probit)

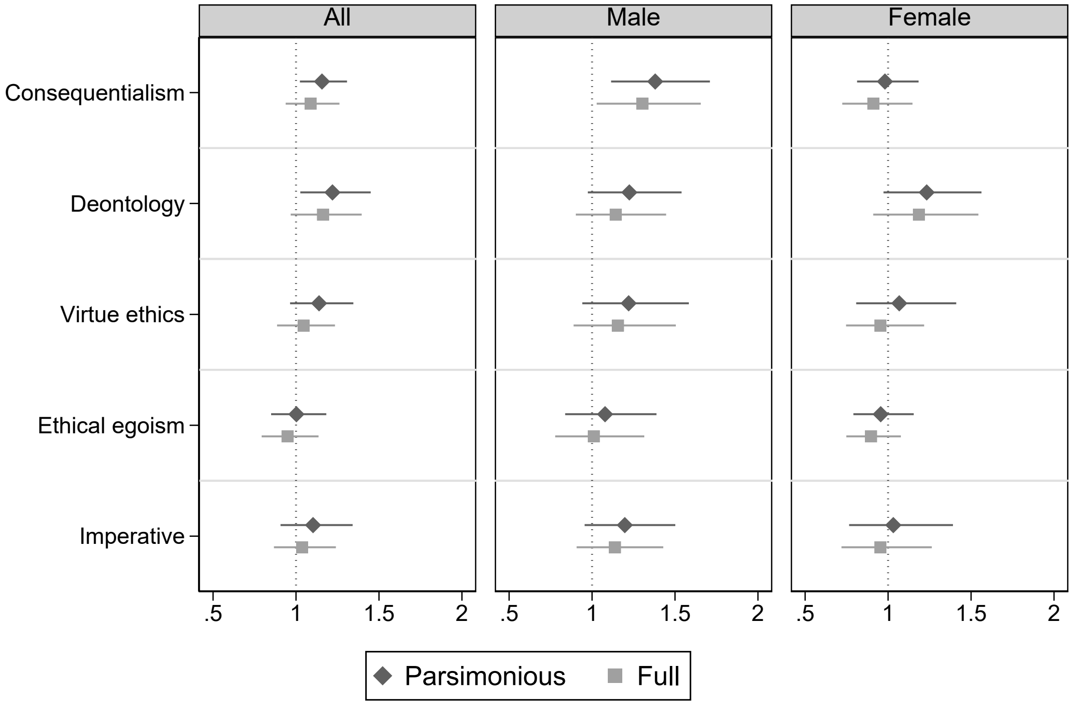

Table SM.4. The impact of moral messages on donations (using Probit)

	(1)	(2)	(3)	(4)	(5)	(6)	(7)	(8)
Consequentialism	0.145**	0.146**	0.085	0.124*	0.088	0.124*	0.082	0.084
	(0.063)	(0.062)	(0.073)	(0.070)	(0.073)	(0.071)	(0.075)	(0.076)
Deontology	0.199**	0.188**	0.155*	0.194**	0.147	0.186**	0.157*	0.150
	(0.088)	(0.088)	(0.092)	(0.087)	(0.094)	(0.089)	(0.092)	(0.093)
Virtue ethics	0.130	0.117	0.064	0.095	0.054	0.086	0.053	0.045
	(0.085)	(0.083)	(0.084)	(0.087)	(0.084)	(0.087)	(0.084)	(0.085)
Ethical egoism	0.003	0.013	-0.040	-0.036	-0.031	-0.027	-0.061	-0.053
	(0.084)	(0.084)	(0.090)	(0.088)	(0.090)	(0.090)	(0.091)	(0.092)
Imperative	0.098	0.094	0.059	0.059	0.060	0.062	0.032	0.036
	(0.010)	(0.099)	(0.095)	(0.098)	(0.094)	(0.097)	(0.092)	(0.091)
Constant	-0.208***	23.04***	0.321***	-0.660***	23.48***	19.29***	-0.544***	20.17***
	(0.058)	(7.482)	(0.123)	(0.206)	(6.957)	(6.774)	(0.182)	(6.434)
Control for demographics	No	Yes	No	No	Yes	Yes	No	Yes
Control for MFQ	No	No	No	Yes	No	Yes	Yes	Yes
Additional controls	No	No	Yes	No	Yes	No	Yes	Yes
N	2426	2426	2426	2426	2426	2426	2426	2426

NOTES: Coefficients from Probit models (standard errors in parentheses) and control for the date of data collection.
$^{*}p < 0.1,^{**}p < 0.05,^{***}p < 0.01$.

Table SM.5. The impact of moral messages on male participants' donations (using Probit)

	(1)	(2)	(3)	(4)	(5)	(6)	(7)	(8)
Consequentialism	0.323***	0.330***	0.267**	0.298**	0.268**	0.302**	0.265**	0.266**
	(0.109)	(0.110)	(0.118)	(0.118)	(0.118)	(0.119)	(0.121)	(0.122)
Deontology	0.203*	0.195*	0.155	0.172	0.142	0.161	0.147	0.133
	(0.117)	(0.115)	(0.123)	(0.117)	(0.121)	(0.113)	(0.124)	(0.120)
Virtue ethics	0.199	0.200	0.144	0.190	0.142	0.191	0.147	0.145
	(0.133)	(0.132)	(0.134)	(0.138)	(0.132)	(0.137)	(0.136)	(0.134)
Ethical egoism	0.0756	0.0816	0.0157	0.0456	0.0161	0.0464	0.0121	0.0112
	(0.129)	(0.129)	(0.135)	(0.132)	(0.135)	(0.132)	(0.133)	(0.134)
Imperative	0.180	0.184	0.141	0.148	0.148	0.157	0.122	0.130
	(0.115)	(0.118)	(0.118)	(0.112)	(0.119)	(0.115)	(0.114)	(0.116)
Constant	-0.358***	15.65*	0.0997	-0.369	19.46**	16.80**	-0.309	18.39**
	(0.0798)	(8.410)	(0.181)	(0.279)	(8.413)	(7.836)	(0.240)	(7.816)
Control for demographics	No	Yes	No	No	Yes	Yes	No	Yes
Control for MFQ	No	No	No	Yes	No	Yes	Yes	Yes
Additional controls	No	No	Yes	No	Yes	No	Yes	Yes
N	1221	1221	1221	1221	1221	1221	1221	1221

NOTES: Coefficients from Probit models (standard errors in parentheses) and control for the date of data collection. $^{*}p < 0.1, ^{**}p < 0.05, ^{***}p < 0.01$.

Table SM.6. The impact of moral messages on female participants' donations (using Probit)

	(1)	(2)	(3)	(4)	(5)	(6)	(7)	(8)
Consequentialism	-0.019	-0.058	-0.080	-0.039	-0.099	-0.065	-0.078	-0.093
	(0.096)	(0.103)	(0.107)	(0.102)	(0.113)	(0.113)	(0.110)	(0.118)
Deontology	0.209*	0.179	0.174	0.235*	0.152	0.210	0.188	0.170
	(0.121)	(0.122)	(0.130)	(0.128)	(0.131)	(0.130)	(0.132)	(0.135)
Virtue ethics	0.065	0.033	-0.017	-0.006	-0.030	-0.019	-0.042	-0.048
	(0.142)	(0.127)	(0.130)	(0.134)	(0.123)	(0.127)	(0.128)	(0.125)
Ethical egoism	-0.046	-0.054	-0.062	-0.106	-0.069	-0.102	-0.108	-0.108
	(0.097)	(0.097)	(0.092)	(0.087)	(0.094)	(0.091)	(0.090)	(0.093)
Imperative	0.031	-0.006	-0.009	-0.0116	-0.031	-0.030	-0.033	-0.048
	(0.152)	(0.154)	(0.145)	(0.149)	(0.147)	(0.152)	(0.140)	(0.144)
Constant	-0.0710	28.46***	0.555***	-1.003***	25.08***	19.06*	-0.812**	18.54**
	(0.091)	(10.43)	(0.213)	(0.272)	(9.430)	(9.858)	(0.353)	(9.236)
Control for demographics	No	Yes	No	No	Yes	Yes	No	Yes
Control for MFQ	No	No	No	Yes	No	Yes	Yes	Yes
Additional controls	No	No	Yes	No	Yes	No	Yes	Yes
N	1205	1205	1205	1205	1205	1205	1205	1205

Notes: Coefficients from Probit models (standard errors in parentheses) and control for the date of data collection. $^{*}p < 0.1, ^{**}p < 0.05, ^{***}p < 0.01$.

Table SM.7. Gender differences in the effect of messages

Dependent variable: Donation to UNICEF		
	(1)	(2)
Consequentialism× male	0.263*<br>(0.143)	0.273*<br>(0.146)
Deontology× male	-0.155<br>(0.117)	-0.203<br>(0.132)
Virtue ethics × male	0.011<br>(0.191)	0.074<br>(0.163)
Ethical egoism× male	-0.007<br>(0.145)	-0.036<br>(0.159)
Imperative × male	0.030<br>(0.132)	0.035<br>(0.127)
Baseline × male	-0.147<br>(0.129)	-0.143<br>(0.125)
Additional controls	No	Yes

Notes: Coefficients from Probit models with two-way fixed effects (standard errors in parentheses) and control for the date of data collection. Additional controls are age, ethnicity, political view, moral conviction and the five MFQ moral values. $^{*}p < 0.1,^{**}p < 0.05,^{***}p < 0.01$.
